# Hydration Mechanisms and Microstructural Evolution of MgO–Metakaolin–Slag Cementitious Materials

**DOI:** 10.1021/acsomega.6c07101

**Published:** 2026-09-08

**Authors:** Junxuan Luo, Xiangyi Cheng, Yongshan Tan

**Affiliations:** School of Civil Engineering and Transportation, 47854 Yangzhou University, Yangzhou 225127, China

## Abstract

This study aims to investigate the hydration process and mechanism of MgO–metakaolin–slag (MMKS) ternary cementitious materials. The effects of different slag (SG) contents (0–40%) and MgO/metakaolin (MK) mass ratios (4:6 and 6:4) on the properties of MMKS cementitious materials were examined. XRD, TG/DTG, IR, solid-state NMR, SEM, and mercury intrusion porosimetry (MIP) were employed to systematically characterize the phase composition, morphological features, and microstructures of the hydration products. The results indicate that when the MgO/MK mass ratio is 6:4 and the SG content is 20%, both the fluidity and compressive strength of the MMKS cementitious paste are significantly improved. Mechanistic analysis shows that SG can be effectively activated in the MMKS system with a high MgO/MK ratio, which not only promotes the polymerization of magnesium silicate hydrate (M–S–H) or magnesium aluminosilicate hydrate (M–A–S–H) gel phases but also facilitates the assembly of active Al^3+^ to form hydrotalcite-like (LDH) phases. These phases act synergistically with the gel phases, thereby markedly enhancing the mechanical performance of the MMKS cementitious materials. Pore structure analysis reveals that an appropriate SG dosage refines the pore size distribution of hardened MMKS cementitious materials, whereas excessive SG incorporation (e.g., 40%) leads to pore coarsening and strength reduction due to a dilution effect. This study elucidates the pathways by which SG enhances the performance of the MgO–MK systems. Through the synergistic design of mixed proportions and dosages, hydration products can be effectively regulated, providing key theoretical and experimental support for the development of high-performance MMKS ternary systems.

## Graphical abstract

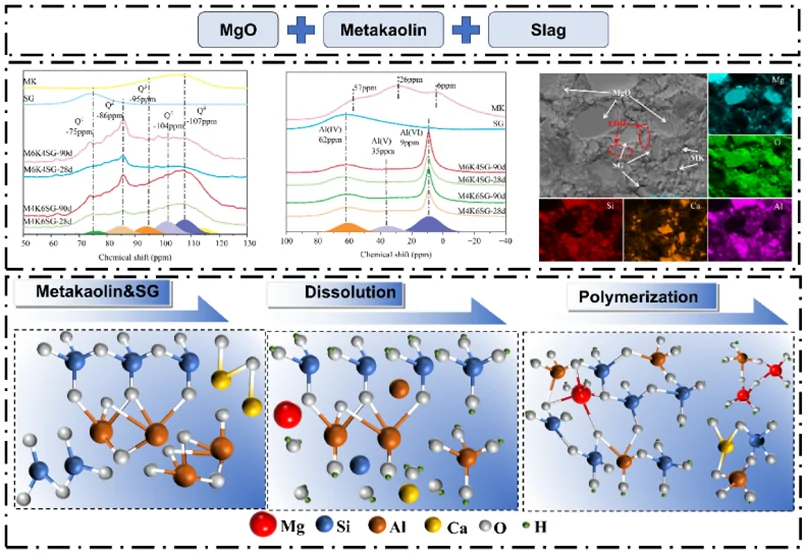

## 1 Introduction

Magnesia-based cementitious materials, a new class of inorganic binders primarily composed of reactive MgO, exhibit distinct advantages in their physical, mechanical, and other engineering properties. They are characterized by rapid hydration, high early strength, no requirement for water curing during setting and hardening, good abrasion resistance and resilience, as well as high mechanical strength.^1–4^ Meanwhile, magnesia-based cementitious materials possess a relatively moderate alkalinity. Compared with the highly alkaline system of traditional Portland cement, their hydration products exhibit lower alkalinity, which not only helps avoid corrosion of auxiliary materials such as fibers in high-alkali environments but has also been widely demonstrated to be suitable for the safe disposal of hazardous wastes.^5–7^ Owing to these unique performance advantages, magnesia-based cementitious materials have attracted increasing attention from both academia and industry in recent years, showing promising prospects in architectural decoration and environmental engineering.^8^

In recent years, research on enhancing the performance of magnesia-based cementitious materials has been deepened. Different types and characteristics of such materials have been developed for various applications. Among them, MgO–metakaolin (MgO–MK), as a novel green magnesia-based binder, exhibits a low heat of hydration, high density, high strength, high volume stability, strong carbonation resistance, and excellent resistance to aggressive environments, demonstrating clear advantages. Shah et al.^9^ found that MgO–SiO_2_ binder systems, especially MK-based systems, tend to produce denser microstructures than PC, with lower porosities and smaller pore sizes. The aluminosilicates in MK can react continuously with brucite over the long term, further improving the microstructure of MgO–SiO_2_ binders. Scott and Shah^10,11^ demonstrated that it is feasible to produce M–S-–H binders using clays and metakaolin with low kaolinite content. Compared with silica fume, clay-based binders show similar or better compressive strengths and lower porosities. Shah et al.^12^ reported that MK blends, together with M–S–H and brucite, form hydrotalcite-like phases. The volumetric characteristics of hydrotalcite contribute to improved mechanical performance and reduced porosity, while aluminum in MK helps enhance the overall properties of MgO–SiO_2_ binders. Compared with ordinary Portland cement, MgO–MK blends exhibit a comparable compressive strength and more favorable pore structure characteristics. Shan and Scott^13^ reported that in MgO–MK systems, hydrotalcite phases form together with brucite and M–S–H, and the volumetric properties of these hydration products help develop a low-porosity microstructure. Compared with MgO-silica fume mixtures, the denser microstructure of MgO–MK mixtures can be clearly observed from compressive strength results at all curing ages. Zhang et al. and Sonat et al.^14,15^ found that MgO–MK systems show significant strength gains between 28 and 90 days, while early-age strength development is comparable to previously reported MgO–silica fume systems. Tan et al.^16^ observed that the main hydration products in MgO–MK systems are brucite, hydromagnesite, and magnesium silicate hydrate (M–S–H) or magnesium aluminosilicate hydrate (M-–A–S–H) gels; increasing the MgO content can lead to the formation of additional hydration products and a more compact microstructure.

These studies confirm that metakaolin is not merely a low-cost substitute for silica fume, but a functional raw material. The aluminum introduced by MK fundamentally alters the phase assemblage and microstructure of the hydration products through the formation of hydrotalcite-like phases, thereby achieving synergistic improvements in mechanical performance, durability, and environmental sustainability. This establishes the foundation for MgO–MK systems as high-performance, low-cost, novel cementitious binders. Current literature mainly focuses on using MK as a core precursor to design and optimize M–S–H-based magnesia cementitious systems. Despite considerable progress, the MgO–MK binary system still faces certain limitations. Its mechanical properties are highly sensitive to the MgO/MK ratio.^16^ A low MgO/MK mass ratio may not fully mobilize the aluminosilicate species in MK,^17^ leading to underutilization of aluminum. In contrast, mineral admixtures such as fly ash and slag, owing to their potential reactivity, are commonly used in high-performance cement and concrete to achieve good durability (e.g., low heat of hydration, high strength, impermeability, and corrosion resistance). However, the compositional design, mechanical properties, hydration process, and micromechanisms of MMKS ternary cementitious systems have not been systematically investigated. Therefore, this study examines the effects of different MgO/MK mass ratios, SG contents, and curing ages on the compressive strength, fluidity, and microstructure of MMKS cementitious materials. X-ray diffraction (XRD), thermogravimetric analysis (TGA), Fourier transform infrared spectroscopy (FTIR), and solid-state nuclear magnetic resonance (NMR) were used to investigate the phase composition and evolution of hydration products in MMKS systems. In addition, scanning electron microscopy (SEM) combined with energy-dispersive X-ray spectroscopy (EDS) and mercury intrusion porosimetry (MIP) were employed to analyze the microstructural characteristics and pore structure evolution of the hydration products. Different from previous studies on binary MgO–MK systems, this work introduces slag as a third component into MMKS systems and systematically varies MgO/MK ratios (4:6 and 6:4) and slag dosages (0–40%) to reveal the synergistic activation mechanism. The novelty lies in multi-scale characterization (NMR, MIP, etc.) that elucidates how a high MgO/MK ratio enables slag to promote both LDH formation and M–(A)–S–H polymerization, leading to a composite gel-crystalline structure with a refined pore system.

## 2 Materials and Methods

### 2.1 Raw Materials

MgO used in this study was supplied by Liaoning Haicheng Jinsha Magnesium Industry Co., Ltd. It was light-burned magnesia calcined at 750–850 °C. The active MgO content of this batch, determined by the hydration method,^18^ was 60.0%. The powder had a fineness of 200 mesh, with a sieve residue of approximately 9%. The metakaolin (MK) was provided by Shanxi SUPER Calcined Kaolin Co., Ltd. The slag (SG) was supplied by Gongyi Longze Water Purification Materials Co., Ltd., with a grade of S95. The laser particle size distributions of MgO, MK, and SG are presented in Figure 1 and Table 1, while Table 2 lists the chemical compositions of MgO, MK, and SG.

**Figure 1:**
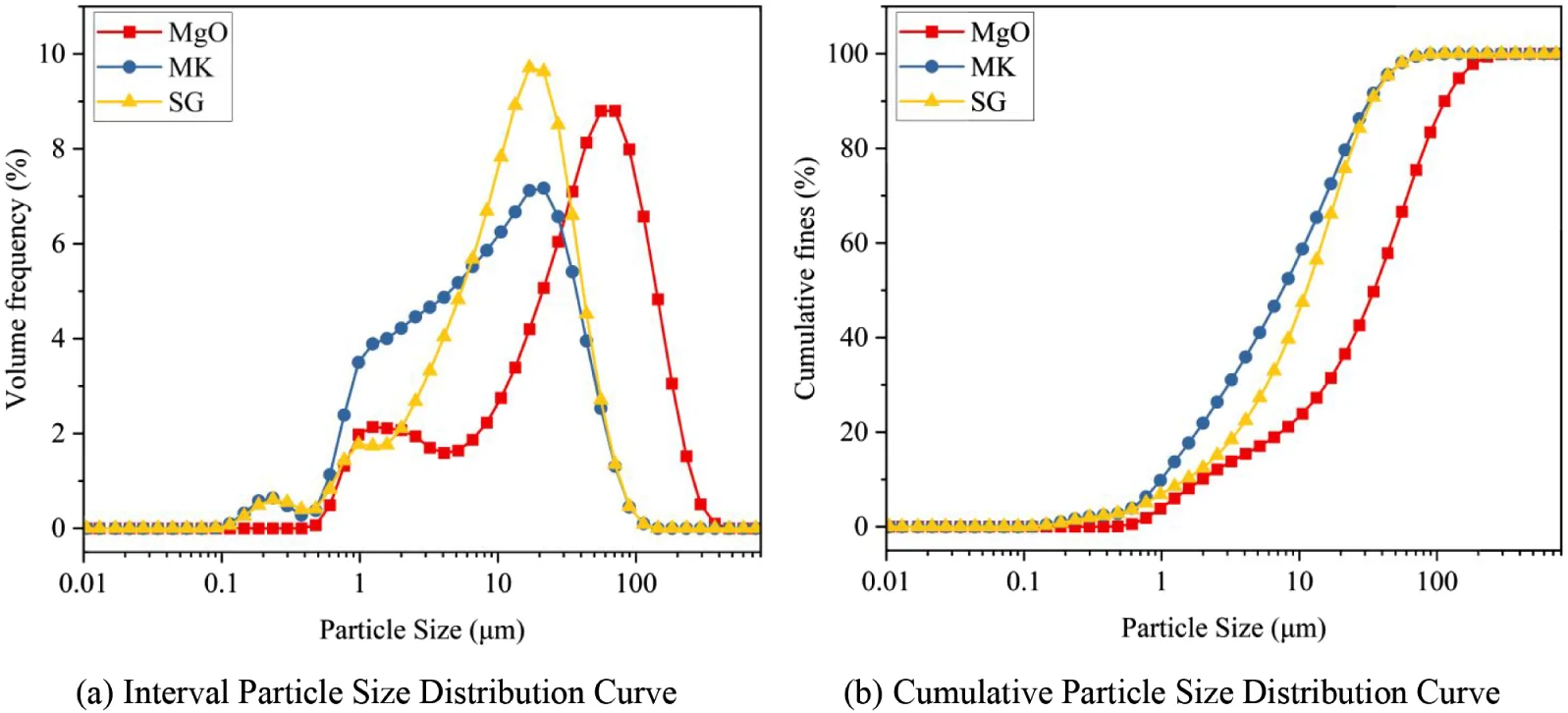
Laser particle-size distribution curves of MgO, MK, and SG.

**Table 1:** Summary of Particle Size and Specific Surface Area Results

**Sample**	**D10 (μm)**	**D50 (μm)**	**D90 (μm)**	**D[4,3] (μm)**	**D[3,2] (μm)**	**SSA** (m^2^/g)	**Span**
MgO	2.19	39.06	127	53.91	7.4	0.3	3.2
MK	1.11	8.43	35.77	14.22	2.63	0.84	4.11
SG	1.69	12.64	37.24	16.76	3.45	0.64	2.81

**Table 2:** Chemical Composition of MgO and MK

**Composition**	**SiO**_**2**_	**Al**_**2**_ **O**_**3**_	**CaO**	**MgO**	**SO**_**3**_	**Fe**_**2**_ **O**_**3**_	**Na**_**2**_ **O**
MgO	2.83	0.27	0.90	95.34	0.30	0.34	-
MK	52.04	45.02	0.54	0.22	-	0.60	0.20
SG	33.50	17.50	35.00	6.01	1.65	1.03	-

### 2.2 Specimen Preparation Method

For the preparation of MMKS cementitious materials, the MgO-to-MK mass ratios were set at 4:6 and 6:4, with a water-to-binder ratio (water to MgO–MK binder, excluding SG) of 0.4. These two ratios were selected based on preliminary experimental screening and previous findings,^16^ which indicated that the M4K6 and M6K4 compositions represent the lower and upper bounds, respectively, of the MgO/MK ratio range that yields reasonable mechanical performance in the binary MgO–MK system. Sodium hexametaphosphate (Na-HMP, analytical grade, purity ≥96%, Sinopharm Chemical Reagent Co., Ltd.) was used as a water-reducing agent and was dissolved in the mixing water prior to addition to the mix, at a fixed dosage of 1% by mass of the MgO–MK binder. SG was incorporated into the MgO–MK cementitious materials using the external addition method, with dosages of 10%, 20%, 30%, and 40% by mass of the MgO–MK binder. According to the designed water-to-binder ratio, the weighed raw materials were first dry-mixed uniformly in a container. Water was then added, followed by low-speed mixing for 30 s and high-speed mixing for 3 min. The freshly prepared slurry was cast into steel molds with dimensions of 40 mm × 40 mm × 40 mm.

The specimens were demolded after 1 day and then cured in a curing room until the designated ages. The selected curing ages of 14, 28, 56, and 90 days were designed to capture both the medium-term strength development (14 and 28 days) and the long-term hydration evolution (56 and 90 days) of MMKS systems, as previous studies on MgO-based binders have shown that significant strength gains and microstructural densification can occur beyond 28 days due to the progressive formation of M–(A)–S–H gels and LDH phases.^13,16^ The curing temperature was maintained at 20 ± 5 °C, and the relative humidity was controlled at 50% ± 5%.

### 2.3 Test Methods

The hydration exothermic performance of the MMKS cementitious system was characterized using an isothermal calorimeter (model TAM AIR-8, TA Instruments, USA) at a fixed temperature of 20 °C. Each specimen was run for a testing period of 72 h. To ensure baseline stability throughout instrument calibration, all experimental procedures were conducted inside a temperature-controlled room. After the cementitious paste was prepared, approximately 5 g of the fresh mixture was quickly pipetted into the base of a glass bottle using a plastic pipette, with care taken not to touch the inner wall, and then the bottle was immediately sealed with a lid. This bottle was gently placed into the measuring channel of the calorimeter, while a reference bottle filled with distilled water was positioned in a separate channel. The measurement was terminated as soon as the recorded heat-flow curve returned to the baseline level.

The compressive strength of the specimens was measured by using a YA-300 microcomputer-controlled automatic compression testing machine manufactured by Changchun Kexin Text Instruments Co., Ltd. Tests were conducted at curing ages of 14, 28, 56, and 90 days with a loading rate of 0.1 MPa/s.

Flowability was evaluated by a minislump test using a geometrically scaled-down Abrams cone with a top diameter of 19 mm, a bottom diameter of 38 mm, and a height of 57 mm. The test was performed on a poly­(methyl methacrylate) plate marked with a 292 cm^2^ grid. A polytetrafluoroethylene slump cone was lifted vertically at a rate of 0.005 m/s to minimize inertial effects. After standing for 1 min, top-view images were captured. The spread area was calculated using calibrated grids and ImageJ software and then converted into an equivalent spread diameter. To reduce the experimental error, three independent batches were prepared for each paste, and three parallel tests were conducted for each batch.

The hydration products of MMKS cementitious materials at different ages were analyzed using a D8 ADVANCE X-ray diffractometer produced by Bruker AXS. Hydration was stopped using anhydrous ethanol. Samples were dried in a vacuum oven for 72 h and then ground in an agate mortar to a particle size below 75 μm. XRD test conditions were as follows: Cu Kα radiation, tube voltage of 40 kV, current of 30 mA, scanning range of 5°–70° (2θ), and scanning rate of 4°/min.

Thermogravimetric analysis (TG) was carried out using a Pyris 1 thermogravimetric analyzer manufactured by PerkinElmer. Samples were heated from room temperature to 1000 °C at a rate of 10 °C/min under a nitrogen atmosphere, with a balance sensitivity of 0.1 mg.

Infrared spectroscopy was conducted using a Cary 610/670 micro-FTIR spectrometer from Varian. The spectral range was 400–4000 cm^–1^, with a resolution better than 0.5 cm^–1^, wavenumber accuracy better than 0.1 cm^–1^, and transmittance accuracy better than 0.1%T. Prior to testing, samples were mixed and ground with KBr at a mass ratio of 1:100 and pressed into pellets.

Micromorphology was observed using a GeminiSEM 300 field-emission scanning electron microscope from Carl Zeiss, with a resolution of 3.5 nm and an accelerating voltage of 20 kV. Samples were prepared as flat slices approximately 5 × 5 × 2 mm^3^, mounted on copper stubs with conductive adhesive, sputter-coated with gold, and examined under vacuum. Elemental mapping was performed in conjunction with an energy-dispersive spectrometer (EDS).

Solid-state nuclear magnetic resonance (NMR) characterization was performed on an AVANCE III 400 MHz wide-bore solid-state NMR spectrometer manufactured by Bruker, with a magnetic field strength of 9.4 T. For ^29^Si and ^27^Al nuclei, the maximum spinning speeds were 7 kHz and 15 kHz, respectively, with an operating frequency of 79.5 MHz. The temperature control range was −150 °C to 180 °C, with an accuracy of ± 0.1 °C.

The pore structure was determined using an AutoPore IV 9500 mercury intrusion porosimeter produced by Micromeritics. The low-pressure station had a degassing vacuum of 5 × 10^–3^ Torr and a pressure range of 3.45–310 kPa, corresponding to measurable pore diameters of 360–3.6 μm. The high-pressure station operated up to 227.527 MPa, enabling measurement of pore sizes down to 5.5 nm.

## 3 Results and Discussion

### 3.1 Flowability

Figure 2 illustrates the effect of different SG contents on the flowability of the MMKS cementitious materials. The results indicate that the incorporation of SG significantly alters the rheological behavior of the paste, and this effect is closely related to the development trend of the compressive strength.

**Figure 2:**
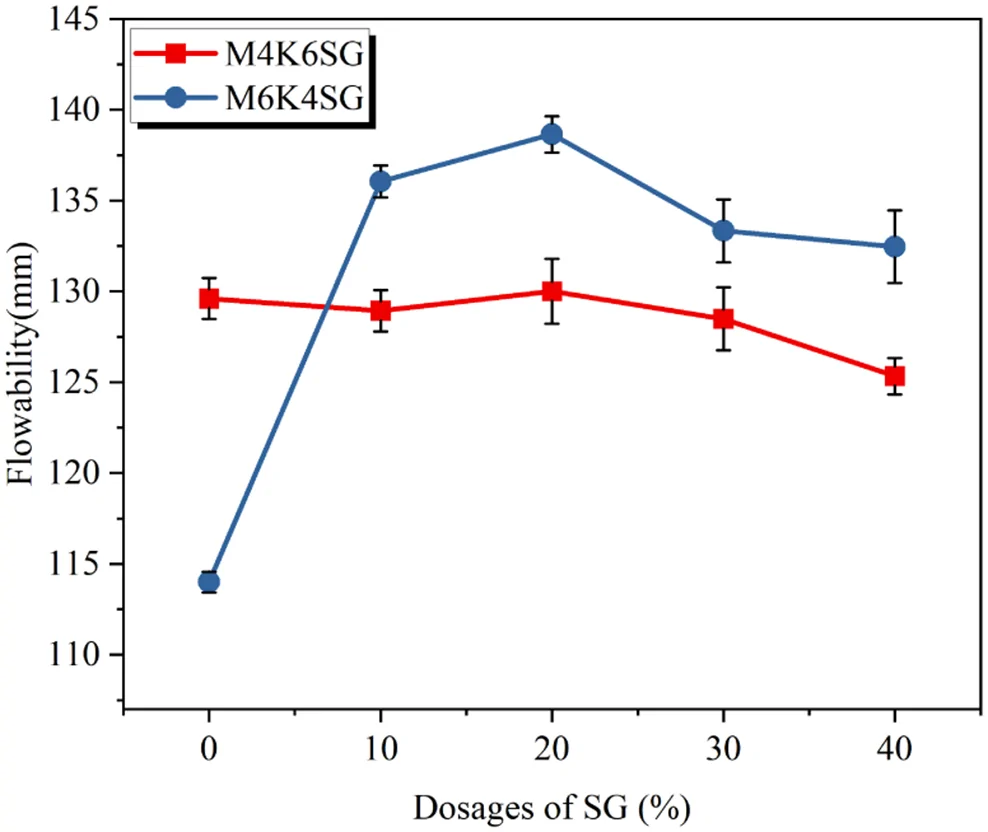
Flowability of MMKS cementitious materials.

For samples with the M4K6 mix ratio, the addition of SG had no significant effect on the workability of MMKS cementitious materials. When the SG content reached 20%, the flowability of the paste was 130.01 mm, representing only a 0.32% increase compared with 129.6 mm for the sample without SG. In contrast, for samples with the M6K4 mix ratio, the improvement in flowability due to SG was more pronounced: with 10% SG, the flowability increased dramatically from 114.00 mm (without SG) to 136.05 mm; when the SG content reached 20%, the flowability peaked at 138.64 mm, corresponding to an increase of 21.61%. Although further increasing the SG content to 30% and 40% led to slight decreases in flowability, to 133.34 mm and 132.45 mm, respectively, these values remained substantially higher than the 114.00 mm observed for the paste without SG, indicating that SG can effectively maintain good workability over a wide range of dosages. It is noteworthy that 20% SG represents a critical point at which the plasticizing effect is fully realized. The improvement in flowability with SG addition may be attributed to its smaller specific surface area (0.64 m^2^/g) compared with MK (0.84 m^2^/g) and its coarser median particle size (12.64 μm versus 8.43 μm for MK), which result in lower water absorption and thus reduce the water demand.

### 3.2 Hydration Heat

Figure 3 presents the hydration heat flow curves and cumulative hydration heat release curves of MMKS cementitious materials with different MgO/MK ratios and SG contents. All specimens exhibit a pronounced initial exothermic peak within 0–2 h after water addition, which is primarily attributed to the wetting and surface physical adsorption of powder particles upon contact with water, together with the exothermic hydration of MgO to form brucite. At the same SG content, the timing and shape of the main exothermic peak differ markedly between the M4K6 and M6K4 groups: the main peak of the M6K4 group consistently appears later than that of the M4K6 group, which may be attributed to the more rapid formation of a brucite coating layer on particle surfaces at higher MgO content, thereby hindering internal ionic diffusion and delaying the onset of the main hydration reaction.

**Figure 3:**
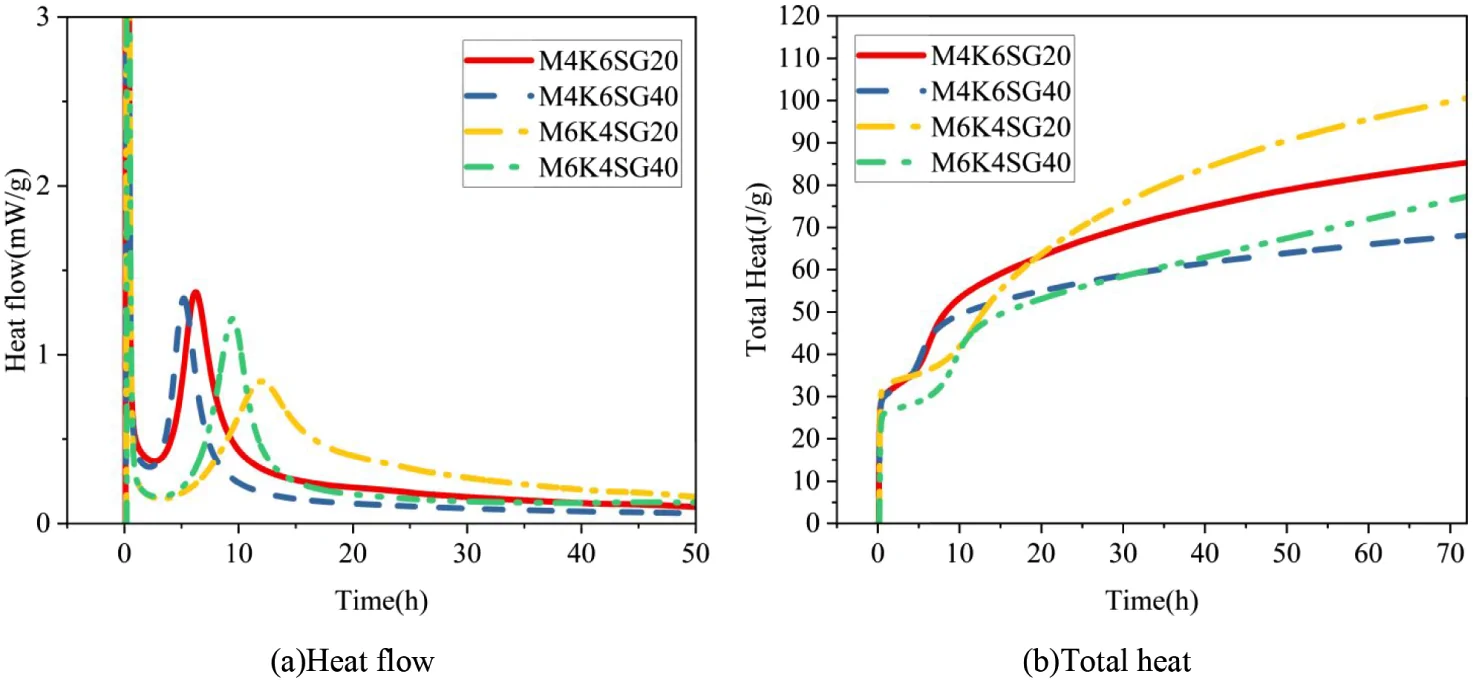
Hydration heat curves of MMKS cementitious materials.

The response of the main exothermic peak intensity to the SG content also differs between the two groups. In the M4K6 group, increasing the SG content from 20% to 40% causes little change in peak intensity, indicating that the increased SG content does not weaken the early-age exothermic intensity of this group. In the M6K4 group, however, increasing the SG content from 20% to 40% markedly increases the peak intensity and shifts its occurrence noticeably earlier, which may be because, at high MgO content, the incorporated SG particles provide additional nucleation sites and a larger specific surface area, promoting more thorough mixing within the system and heterogeneous nucleation of hydration products.

The cumulative hydration heat curves (Figure 3b) show a final heat release ranking of M6K4SG20 > M4K6SG20 > M6K4SG40 > M4K6SG40. Under the same MgO/MK ratio, increasing the SG content from 20% to 40% reduces the total heat release in both groups, consistent with a dilution effect: a higher proportion of SG lowers the effective content of MgO and MK per unit mass of cementitious material, thereby limiting the short-term reactive capacity of the system.

### 3.3 Compressive Strength

Figure 4 illustrates the effect of varying MgO/MK mass ratios and SG contents on the compressive strengths of MMKS cementitious materials at different curing ages.

**Figure 4:**
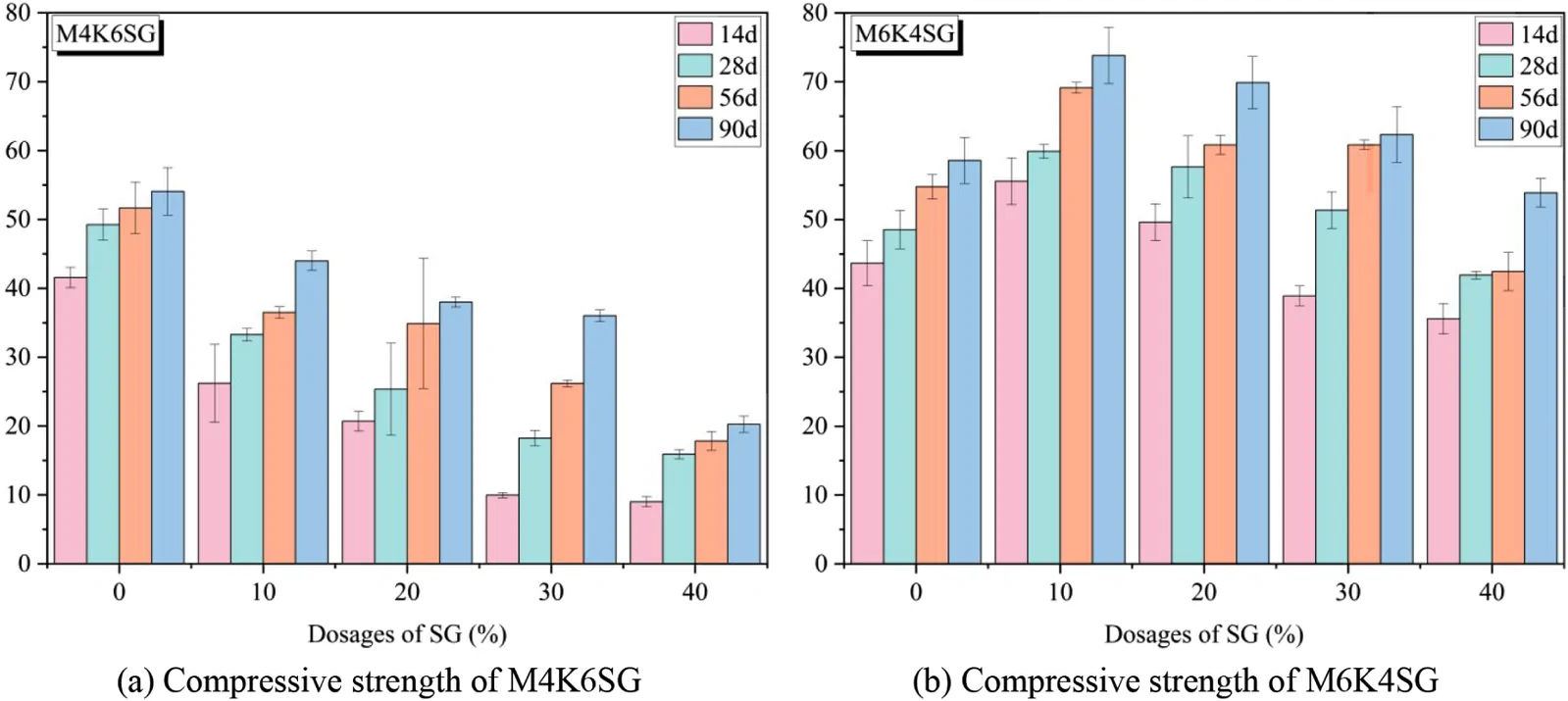
Compressive strength of MMKS cementitious materials.

Overall, the compressive strength of MMKS cementitious materials increased continuously with curing time but generally decreased with increasing SG content. For example, in samples with the M4K6 mix ratio, the 28-day compressive strength was 49.25 MPa without SG. When the SG content was increased to 20%, the 28-day compressive strength decreased to 25.35 MPa, showing a monotonic decline with increasing SG dosage. In contrast, for samples with the M6K4 mix ratio, the addition of SG significantly enhanced the compressive strength of the paste: with 10% SG, the 28-day compressive strength reached a peak of 59.90 MPa; with 20% SG, the 28-day strength remained high at 57.64 MPa, markedly higher than that of the sample without SG and only 3.77% lower than the peak strength.

These results indicate that the compressive strength of MMKS cementitious materials is highly dependent on the composition of the matrix. In low MgO/MK systems (M4K6), MK and SG could not be fully activated. However, a moderate SG addition (10–20%) in high MgO/MK systems significantly promoted strength development. This can be attributed to two main factors. First, under the alkaline environment provided by the hydrolysis of MgO (pH ≈ 10.5), the glassy phase of slag undergoes depolymerization and dissolution, releasing Ca^2+,^ Al^3+^, and silicate species.^19^ These reactive species subsequently participate in the formation of calcium (alumino) silicate hydrate (C-(A)–S–H) gels. The formation mechanism involves the condensation of dissolved silicate species (derived from slag and MK) with Ca^2+^ ions released from slag, forming a Ca-rich gel phase that coexists with the Mg-dominated M–(A)–S–H gel. This is fundamentally different from Portland cement hydration, where C–S–H forms directly from the hydration of C_3_S and C_2_S; here, C–(A)–S–H formation relies on the alkali-activated dissolution of slag’s amorphous glass network in the MgO-generated alkaline environment, without the presence of Portlandite (Ca­(OH)_2_). The C–(A)–S–H gels intermix with the M–(A)–S–H gels derived from MgO and metakaolin, contributing to a denser microstructure and improved mechanical strength of the MMKS ternary system. This process is fundamentally different from the classical hydration of discrete C_2_S in Portland cement, as slag activation primarily relies on the breakdown of its amorphous glass network rather than the hydration of crystalline silicate phases. Second, in high MgO/MK systems, the elevated MgO content undergoes continuous hydrolysis, releasing both Mg^2+^ and a large quantity of OH^–^, thereby substantially increasing the alkalinity of the system. This highly alkaline environment effectively activates the reactive silicate and aluminate components in MK and SG, leading to the formation of magnesium silicate hydrate (M–S–H) or aluminum-containing magnesium silicate hydrate (M-A–S–H) gels. These gels interweave and coexist with the previously formed C–(A)–S–H gels, collectively forming a dense composite gel structure that synergistically promotes the overall strength development of the system.

However, when the SG content exceeds a threshold, the compressive strength decreases significantly. A 20% SG dosage achieves relatively high strength, and considering the results on workability, subsequent studies focus on the effects of 20% SG on the hydration process, microstructure, and hydration mechanisms of MMKS cementitious materials.

### 3.4 Phase Analysis

#### 3.4.1 XRD

In this study, phase analysis was conducted on MMKS cementitious materials with SG contents of 20% and 40% and different MgO/MK mass ratios, as shown in Figure 5. The diffraction patterns display characteristic peaks of MgO, Mg­(OH)_2_, and SiO_2_. The MgO peaks correspond to unreacted magnesium oxide, while the SiO_2_ peaks mainly originate from quartz impurities in the raw materials. The persistence of MgO peaks in the XRD patterns, even at 90 days of curing, does not necessarily indicate that MgO is in excess from a stoichiometric perspective. This observation can be explained from two aspects. First, the MgO used in this study is light-burned magnesia with an active MgO content of approximately 60% (as determined by the hydration method), meaning that a considerable portion of the MgO particles possess relatively low reactivity and are difficult to fully participate in chemical reactions within a short period. The remaining less-reactive fraction persists as residual periclase in the matrix. Second, the MgO–MK system exhibits relatively low alkalinity (pH ≈ 10.5, maintained by brucite saturation), which results in slower overall reaction kinetics compared to highly alkaline Portland cement systems. The dissolution of MgO and the subsequent pozzolanic reactions with MK and SG proceed gradually over time, so a substantial amount of MgO remains unreacted at the tested curing ages. With prolonged curing time and under adequate moisture conditions, the residual MgO is expected to continue hydrating progressively, as evidenced by the continuous strength gain observed from 28 to 90 days. This gradual consumption of MgO over time is consistent with the long-term nature of MgO-based cementitious systems reported in the literature.^8^

**Figure 5:**
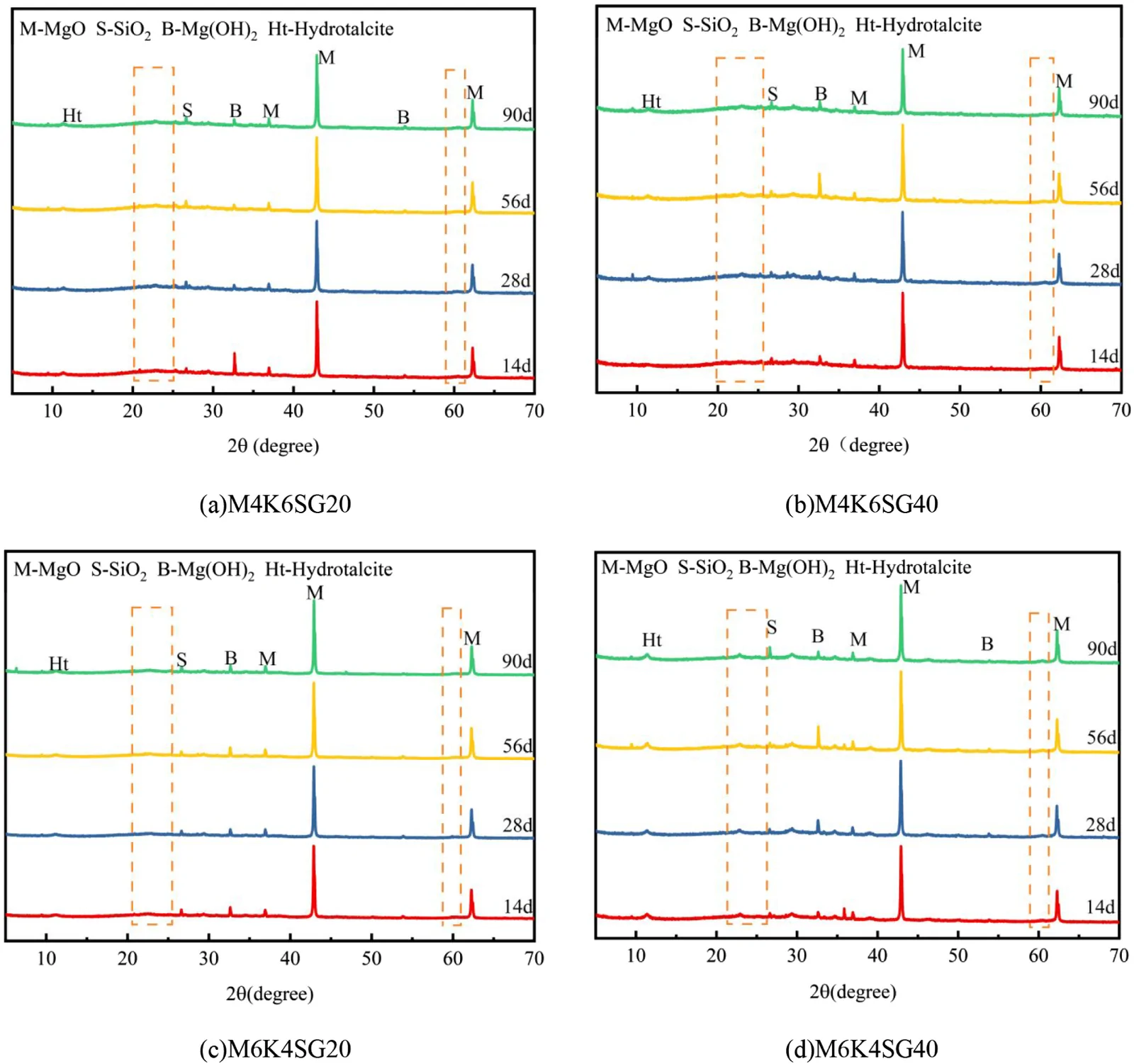
X-ray diffraction patterns of MMKS cementitious materials.

The diffraction patterns display characteristic peaks of MgO, Mg­(OH)_2_, and SiO_2_. The MgO diffraction peaks indicate the presence of unreacted periclase (MgO) that remained after hydration, while the SiO_2_ peaks mainly originate from quartz impurities in the raw materials. The persistence of MgO peaks in the XRD patterns, even at 90 days of curing, does not necessarily indicate that MgO is in excess from a stoichiometric perspective. Rather, it reflects the limited dissolution rate of light-burned MgO under ambient curing conditions; previous studies have shown that complete MgO conversion in magnesia-based cementitious systems typically requires extended curing times or elevated temperatures. In the present system, the residual MgO serves as an internal reservoir that sustains a moderately alkaline pH (∼10.5) over the long term, which is beneficial for the continuous activation of MK and SG. This is consistent with the progressive increase in compressive strength observed from 28 to 90 days.

Notably, in the MMKS samples containing SG, a new characteristic diffraction peak was observed at around 2θ ≈11.2° ^20–22^ (PDF# 56-0956), which can be clearly attributed to layered double hydroxides (LDH), a hydrotalcite-like phase. The formation of this hydrotalcite-like phase has been reported in alkali-activated cementitious systems,^23^ particularly in systems containing reactive aluminum sources (such as SG) and magnesium sources.^24^ The mechanism involves Al^3+^ ions released from the hydrolysis of MK and SG combining with Mg^2+^ in the alkaline environment, with interlayers occupied by anions and water molecules.

The formation of this new phase directly explains, at the phase level, why MMKS samples (especially under M6K4 conditions) with 20% SG can still exhibit excellent mechanical performance. The generation of LDH has a crucial positive impact on material properties: on the one hand, its layered structure effectively fills pores, densifying the microstructure; on the other hand, it can immobilize free Al^3+^ and OH^–^, optimizing the pore solution chemistry and significantly enhancing the dimensional stability of the material.^20^ This provides key scientific evidence for the improvement of compressive strength in MMKS cementitious materials.

#### 3.4.2 TGA

In this study, thermogravimetric (TG) and differential thermogravimetric (DTG) analyses were performed on MMKS cementitious materials with SG contents of 20% and 40% and different MgO/MK mass ratios, as shown in Figure 6. The characteristic peaks in the DTG curves clearly reveal the decomposition processes of various hydration products within specific temperature ranges, providing key evidence to elucidate the role of SG from the perspective of its thermal stability.

**Figure 6:**
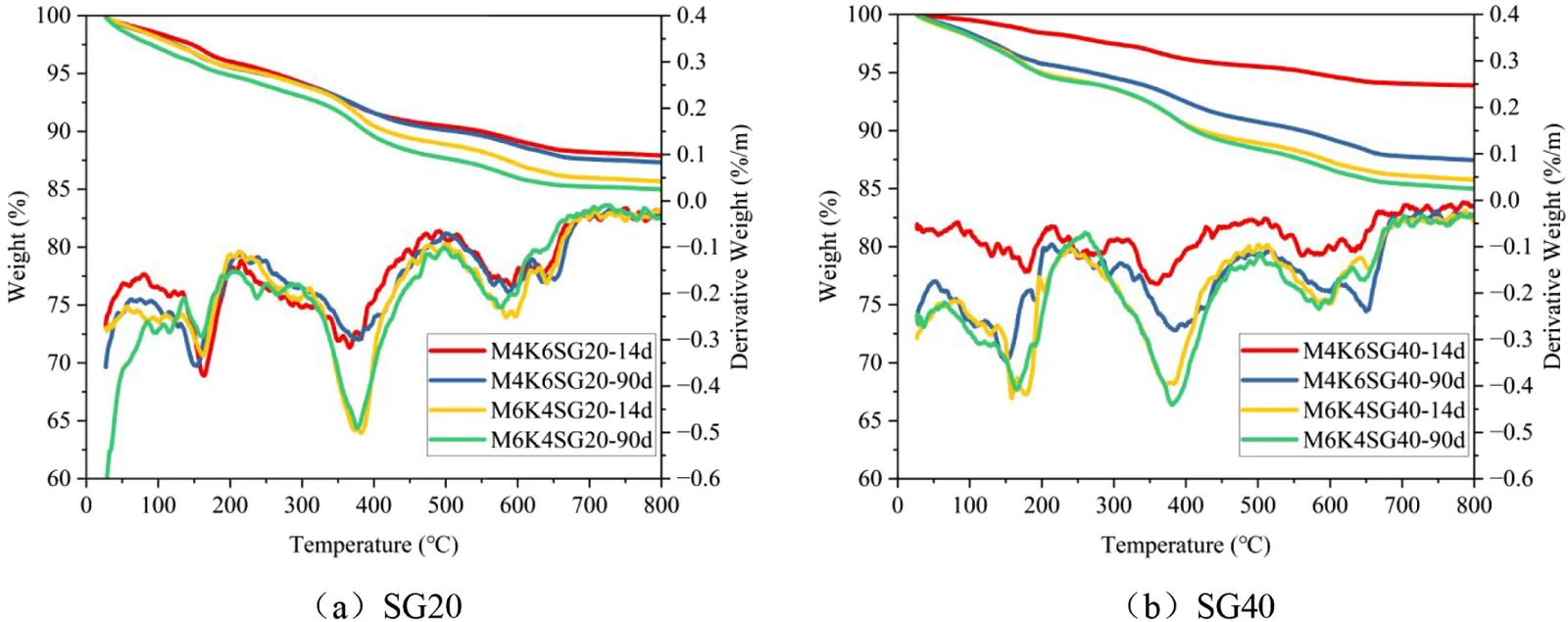
Thermogravimetric analysis results of MMKS cementitious materials at different curing ages.

The thermal decomposition profiles of MMKS cementitious materials show features closely related to the formation of the hydrotalcite-like phase (LDH). The DTG curves exhibit three main stages of weight loss:^20,25,26^ First stage (100–200 °C): the weight loss is attributed to the removal of interlayer-adsorbed water in the LDH phase or partial dehydration of some hydration products. Second stage (300–550 °C): the decomposition peaks are mainly associated with the dehydroxylation of brucite and the continued dehydration of M–(A−)­S–H. Notably, a dominant, continuous mass loss peak appears in the broad range of 400–550 °C, which clearly corresponds to the progressive dehydroxylation of the LDH phase. This feature is characteristic of SG-containing systems and strongly corroborates the LDH peak observed around 2θ ≈11.2° in the XRD patterns. A minor peak near 530 °C may result from the loss of strongly bound coordinated water in M–A–S–H. Third stage (550–800 °C): weight loss in this stage is attributed to the thermal decomposition of various carbonate phases, such as magnesium carbonate formed via raw material carbonation and calcite.

For samples cured for 90 days, the weight loss in the first, second, and third stages was as follows: M4K6SG20, 2.59%, 4.42%, and 2.31%; M4K6SG40, 2.63%, 4.40%, and 2.70%; M6K4SG20, 3.07%, 6.19%, and 2.80%; and M6K4SG40, 3.30%, 5.92%, and 2.71%.

It can be observed that in samples with the M6K4 mix ratio, the weight loss in the second stage was significantly higher than that in M4K6 samples, indicating a higher proportion of LDH and M–(A−)­S–H in M6K4 systems. These findings are consistent with the previously discussed compressive strength and XRD analysis results.

#### 3.4.3 FTIR

FTIR analysis was conducted on MMKS cementitious materials with 20% SG at different MgO/MK mass ratios, and the results are shown in Figure 7. The positions and intensity variations of characteristic absorption bands reveal the regulatory effect of SG on the silicate network structure, the aluminum coordination environment, and the hydration process.

**Figure 7:**
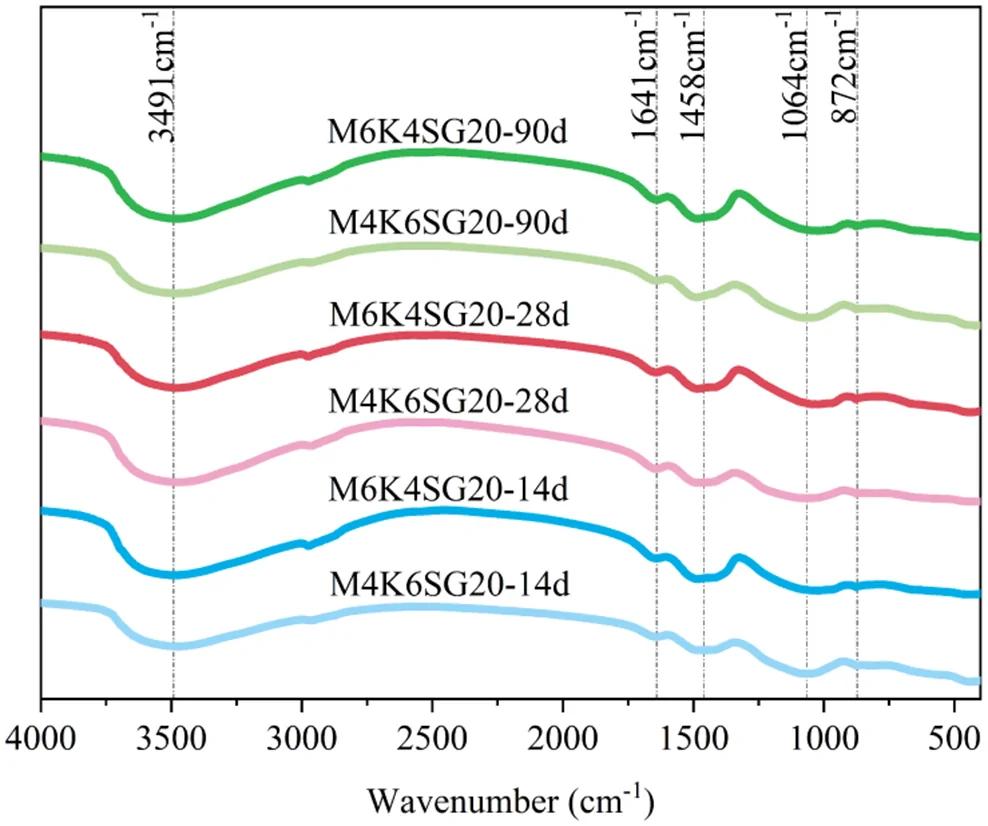
FTIR spectra of MMKS cementitious materials at different curing ages.

The most prominent spectral features of MMKS cementitious materials containing SG are associated with the participation of aluminum species and the formation of new phases. Firstly, broad absorption bands appear at approximately 3491 cm^–1^ and 1641 cm^–1^, corresponding to the stretching vibration of O–H/Mg–OH and the bending vibration of H–O–H, respectively, which are typical indicators of bound water in hydration products such as brucite and M–A–S–H gel.^27,28^ Secondly, distinct absorption bands observed at around 1064 cm^–1^ indicate the formation of magnesium silicate hydrate (M–S–H).^29^

Notably, two distinct absorption bands appear at approximately 1458 cm^–1^ and 872 cm^–1^. The band near 1458 cm^–1^ is assigned to the asymmetric stretching vibration (ν_3_) of CO_3_^2–^, while the band at 872 cm^–1^ corresponds to the out-of-plane bending vibration (ν_2_) of CO_3_^2–^.^30,31^ The appearance of these characteristic carbonate bands may arise from two possible sources: first, slight surface carbonation of the sample during preparation or testing; second, the presence of CO_3_^2–^ as interlayer charge-balancing anions in the hydrotalcite-like (LDH) phase, which is commonly observed in alkali-activated Mg–Al systems.^32^ The efficient utilization of aluminum species in the MMKS system promotes the transformation from primarily silicate tetrahedral-based gels (M–A–S–H) to composite gels enriched with Mg, Al, and Si, as well as the formation of LDH crystalline phases.

#### 3.4.4 ^27^Al MAS NMR

^27^Al solid-state NMR analysis was conducted on MMKS cementitious materials with identical SG content under different MgO/MK mass ratios, and the spectra were quantitatively deconvoluted, as shown in Figure 8 and Table 3. By tracking the relative content changes of Al in different coordination states, we can elucidate the reaction mechanism of SG in the hydration process from the local chemical environment.

**Figure 8:**
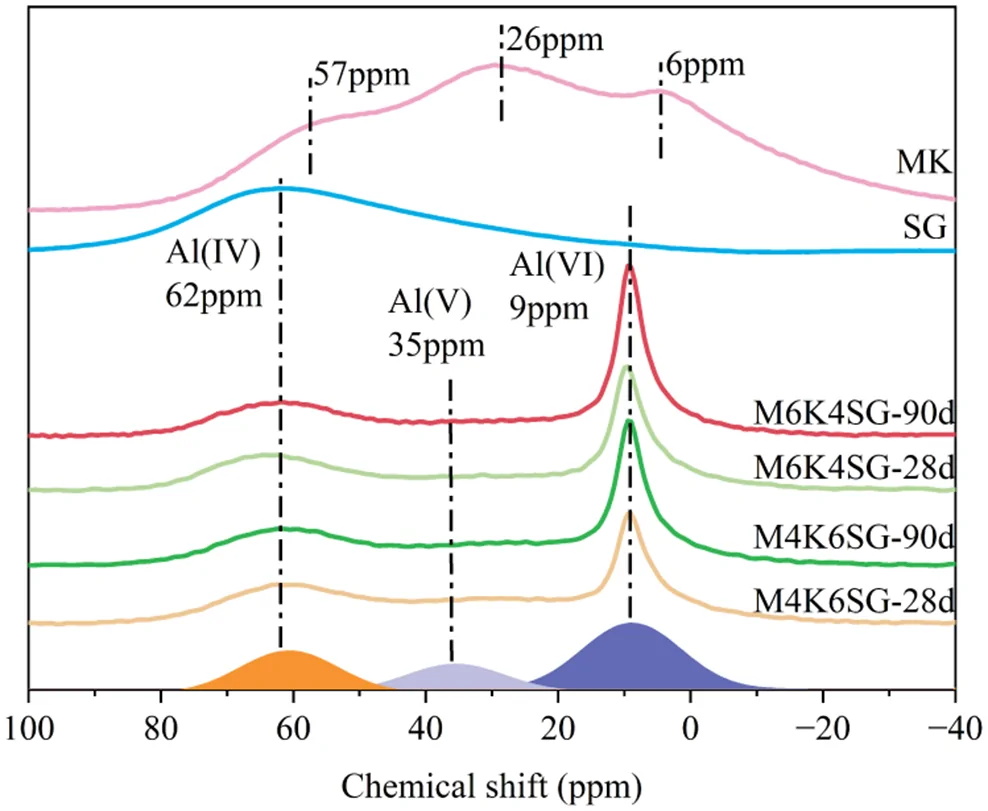
^27^Al solid-state NMR spectra of MMKS cementitious materials at different curing ages.

**Table 3:** Quantitative Deconvolution Results of ^27^Al NMR Spectra for MMKS Cementitious Materials at Different Curing Ages

	Al(IV)		Al(V)		Al(VI)	
Simple ID	Center (ppm)	Area (%)	Center (ppm)	Area (%)	Center (ppm)	Area (%)
M4K6SG (28 days)	60.75	31.18	35.61	20.52	8.86	48.30
M4K6SG (90 days)	60.53	26.99	34.07	16.98	9.06	56.03
M6K4SG (28 days)	62.58	29.51	35.99	13.16	8.55	57.34
M6K4SG (90 days)	61.62	22.56	26.59	11.08	8.91	66.35

The overall features of the ^27^Al NMR spectra indicate that all samples exhibit three characteristic resonance peaks at approximately 9, 35, and 62 ppm, corresponding to six-coordinated aluminum [Al­(VI)], five-coordinated aluminum [Al­(V)], and four-coordinated aluminum [Al­(IV)], respectively.^33^ The Al­(VI) peak near 9 ppm is the most intense and sharpest, indicating that aluminum in the hardened MMKS cementitious material matrix predominantly exists in an octahedral environment. Compared with the raw MK spectrum, aluminum in MK primarily appears near 57 ppm (Al­(IV)), 26 ppm (Al­(V)), and 6 ppm (Al­(VI));^34^ in contrast, aluminum in raw SG mainly appears near 62 ppm (Al­(IV)). Systematic shifts of Al­(VI) and Al­(IV) peaks after hydration reflect significant changes in the local chemical environment as aluminum is released from raw materials and incorporated into newly formed hydration products.^35^

Quantitative deconvolution data reveal clear evolutionary trends. Raw MK is dominated by Al­(V), while raw SG is dominated by Al­(IV). In hydrated and hardened MMKS cementitious materials, the intensities of Al­(IV) and Al­(V) peaks are significantly reduced, indicating that aluminum from both sources transforms into a more stable octahedral environment. The hydrolysis of MgO facilitates the activation of MK and SG, breaking Si–O–Si and Si–O–Al bonds in MK and forming M-(A-)­S–H gel.^16^ Moreover, the presence of MgO accelerates SG hydration, leading to further Al–O bond cleavage.^25^ Quantitative analysis shows that after 28 days of curing, the Al­(VI) content in M6K4 samples is 57.34%, whereas in M4K6 samples it is 48.30%. The Al­(IV) resonance at 62 ppm is attributed to M–A–S–H, while Al­(VI) signals correspond to LDH or Al­(OH)_3_.^25^ Thus, M6K4 samples contain a higher proportion of LDH/Al­(OH)_3_. However, it should be noted that the increase in Al­(VI) content cannot be exclusively attributed to LDH formation, as Al­(OH)_3_ also exhibits six-coordinated aluminum and may contribute to the resonance near 9 ppm. In the present system, the alkaline pH (approximately 10.5) maintained by Mg­(OH)_2_ saturation^36,37^ is not thermodynamically favorable for the precipitation of pure Al­(OH)_3_, which typically forms at pH > 11.5–12.^38^ The pH of MgO–MK systems has been reported to be in the range of 10–10.5, which is consistent with the pH controlled by residual brucite.^36^ At this pH level, the concomitant presence of Mg^2+^ in the pore solution strongly promotes the incorporation of Al into the LDH structure rather than the formation of discrete Al­(OH)_3_ precipitates.^23^ Therefore, the observed increase in Al­(VI) is predominantly assigned to the LDH phase, with only a minor possible contribution from Al­(OH)_3_, if any.

With increasing curing time, both the signal intensity and relative content of the Al­(VI) peak in MMKS samples significantly increase. These observations are consistent with XRD and TG/DTG analyses, which indicate substantial formation of hydrotalcite-like (LDH) phases, as Al^3+^ in LDH layers adopts a characteristic octahedral ([Al­(OH)_6_]^3–^) coordination^39^ Therefore, the increase in Al­(VI) content serves as direct evidence of extensive LDH formation,^40^ and a high MgO/MK mass ratio further promotes the generation of the LDH phase.

A quantitative correlation can be established between the Al­(VI) content derived from ^27^Al NMR and the compressive strength of MMKS cementitious materials. The M6K4SG sample at 28 days exhibits 57.34% Al­(VI) and achieves a 28-day compressive strength of 57.64 MPa, whereas the M4K6SG sample exhibits only 48.30% Al­(VI) with a correspondingly lower strength of 25.35 MPa. At 90 days, the Al­(VI) content of the M6K4SG sample increases to 66.35%, accompanied by a further increase in compressive strength to 69.88 MPa. In contrast, the M4K6SG sample shows an Al­(VI) content of 56.03% at 90 days with a compressive strength of 37.99 MPa. This strong positive correlation (*R* ^2^ = 0.97) indicates that the formation of six-coordinated aluminum species—predominantly LDH phases—is directly associated with the enhancement of mechanical performance. The higher Al­(VI) content in M6K4 systems thus serves as a quantitative predictor of superior mechanical properties, confirming that the incorporation of Al^3+^ into LDH contributes to microstructural densification.

#### 3.4.5 ^29^Si MAS NMR

^29^Si solid-state NMR analysis was conducted on MMKS cementitious materials with identical SG content under different MgO/MK mass ratios, and quantitative data were obtained through deconvolution, as shown in Figure 9 and Table 4. The coordination environment of silicon (Si), denoted as *Q* ^*n*^ (where *n* represents the number of bridging Si atoms), directly reflects the degree of polymerization and structural order of the M–(A−)­S–H gel.

**Figure 9:**
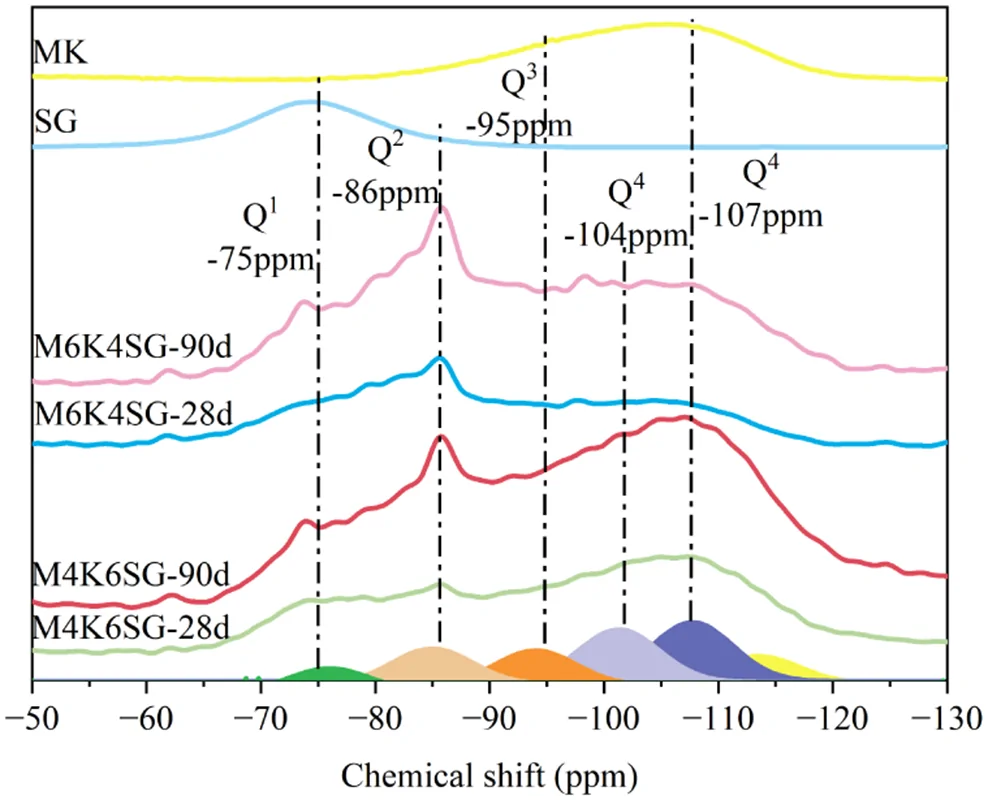
^29^Si solid-state NMR spectra of MMKS cementitious materials at different curing ages.

**Table 4:** Quantitative Deconvolution Results of ^29^Si NMR Spectra for MMKS Cementitious Materials at Different Curing Ages

	*Q* ^1^		*Q* ^2^		*Q* ^3^		*Q* ^4^	
Simple ID	Center (ppm)	Area (%)	Center (ppm)	Area (%)	Center (ppm)	Area (%)	Center (ppm)	Area (%)
M4K6SG (28 days)	–76.42	10.06	–85.39	15.32	–94.43	15.05	–108.14	22.25
					–99.70	22.61	–113.95	11.72
M4K6SG (90 days)	–75.19	5.15	–85.93	17.79	–93.48	13.23	–105.86	20.47
					–99.78	16.88	–111.58	16.78
M6K4SG (28 days)	–74.83	7.28	–85.45	24.95	–92.08	10.82	–103.04	12.17
					–97.80	11.55	–107.71	11.79
M6K4SG (90 days)	–75.66	6.41	–85.77	23.59	–92.32	12.671	–103.78	12.96
					–98.32	13.67	–108.92	12.62

The ^29^Si NMR spectra of all hardened cementitious samples show peaks primarily in the chemical shift range of −75 ppm to −110 ppm. By comparison, raw MK exhibits a single, highly polymerized *Q* ^4^ resonance at approximately −107 ppm, while raw SG shows a *Q* ^1^ resonance near −75 ppm.^25^ In the hardened samples, SiO_4_ tetrahedra corresponding to different polymerization states were observed, including *Q* ^1^ (−75 ppm), *Q* ^2^ (−86 ppm), *Q* ^3^ (−95 ppm), and *Q* ^4^ (−104 to −107 ppm).^26,41^ *Q* ^1^, *Q* ^2^, and *Q* ^3^ signals originate from M–S–H, M–(A−)­S–H, or carbonate-associated M–S–H resonances.^25^ The coexistence of multiple peaks confirms that incorporation of MK and SG produces M–A–S–H gels with layered to partially three-dimensional, cross-linked complex structures.^33,42^

The presence of *Q* ^1^, *Q* ^2^, and *Q* ^3^ units indicates that the resulting M–(A−)­S–H gel possesses a layered molecular configuration. The relative content of *Q* ^1^ units reflects the dispersion of the gel network, with higher values typically indicating lower polymerization, while *Q* ^2^ units are closely related to the formation and growth of chain-like structures.^16^

Comparison of ^29^Si MAS NMR spectra between raw MK, SG, and hardened MMKS cementitious materials shows a significant reduction in the intensities of the *Q* ^1^ and *Q* ^4^ peaks in MMKS samples. This indicates that under continuous Mg^2+^ release from MgO hydrolysis, active SiO_2_ in MK and SG gradually dissolves and participates in the formation of M–(A−)­S–H gels. Moreover, the evolution of the silicate network varies notably with different MgO/MK ratios. For M4K6 samples, a distinct *Q* ^4^ peak remains near −107 ppm, indicating the presence of unreacted crystalline SiO_2_. In contrast, in M6K4 samples, the intensity of the *Q* ^4^ peak was significantly reduced. This key difference suggests that, under high MgO/MK conditions, the rapid formation of Mg­(OH)_2_ markedly increases pore solution concentration, greatly promoting the dissolution of amorphous SiO_2_.^43,44^ The dissolved reactive Si is effectively incorporated into the hydration products, resulting in a substantial decrease in the residual crystalline SiO_2_ signal.

Overall, ^29^Si solid-state NMR analysis demonstrates that the incorporation of SG, in synergy with an appropriate MgO/MK ratio (M6K4), effectively enhances the polymerization state of the silicate network in the MgO–MK systems. Under these conditions, SG promotes SiO_2_ dissolution and gel polymerization, forming a highly polymerized M–A–S–H gel network. This atomic-scale structural evolution, together with the aluminum incorporation into LDH revealed by ^27^Al NMR, provides the chemical basis for the excellent macroscopic mechanical performance of the MMKS cementitious materials.

To further quantify the degree of silicate polymerization, the *Q* ^2^/(*Q* ^1^+*Q* ^2^) ratio and the mean chain length (MCL) of the silicate tetrahedra were calculated based on the deconvoluted ^29^Si NMR data. The *Q* ^2^/(*Q* ^1^ + *Q* ^2^) ratio serves as an indicator of chain elongation in M–(A)–S–H gels, while the MCL was estimated as MCL = 2*Q* ^2^/(*Q* ^1^ + *Q* ^2^). For the M6K4SG sample at 90 days, these values reach 0.786 and 9.36, respectively, significantly higher than those of the M4K6SG sample at 28 days (0.604 and 5.04), demonstrating a more highly polymerized silicate network under high MgO/MK conditions. With an increasing curing time from 28 to 90 days, the M6K4SG sample shows a moderate increase in both the *Q* ^2^/(*Q* ^1^+*Q* ^2^) ratio (from 0.774 to 0.786) and MCL (from 8.85 to 9.36), while the M4K6SG sample exhibits a more pronounced increase (from 0.604 to 0.775 and from 5.04 to 8.91), indicating that the M4K6 system also undergoes progressive polymerization over time, albeit starting from a substantially lower baseline. This enhanced polymerization is closely correlated with the compressive strength development: the M6K4SG sample, with the highest *Q* ^2^/(*Q* ^1^+*Q* ^2^) ratio (0.786), exhibits a 90-day compressive strength of 69.88 MPa, whereas the M4K6SG sample, with a lower ratio (0.604), achieves only 25.35 MPa at 28 days. Such quantitative correlation between NMR-derived structural parameters and mechanical performance reinforces the conclusion that the polymerization of M–(A)–S–H networks is a key contributor to the superior strength of M6K4 systems.

### 3.5 Microstructure and Elemental Mapping

To visually reveal the effect of SG on the microstructural morphology of MMKS cementitious materials, backscattered electron (BSE) imaging and corresponding elemental mapping analyses were conducted on high SG content (40%) MMKS samples with the M6K4 mix ratio, as shown in Figure 10. The M6K4SG40 composition was specifically selected for this microstructural characterization on the basis of two considerations. First, the M6K4 mix ratio yields the optimal mechanical performance among the tested formulations, indicating that under this condition, both MK and SG can be effectively activated, leading to a more favorable microstructure. Second, while we initially attempted to examine the morphological differences induced by varying SG contents using SEM, it proved difficult to distinguish systematic variations in micromorphology across different SG dosages, as SEM is inherently a qualitative rather than quantitative technique for phase identification. Therefore, we selected the M6K4SG40 composition as a representative case to provide clear visual evidence of the microstructural features and elemental distribution in the MMKS system rather than to perform a quantitative comparative analysis across different SG dosages. The pore structure evolution with varying SG contents was systematically characterized by MIP analysis.

**Figure 10:**
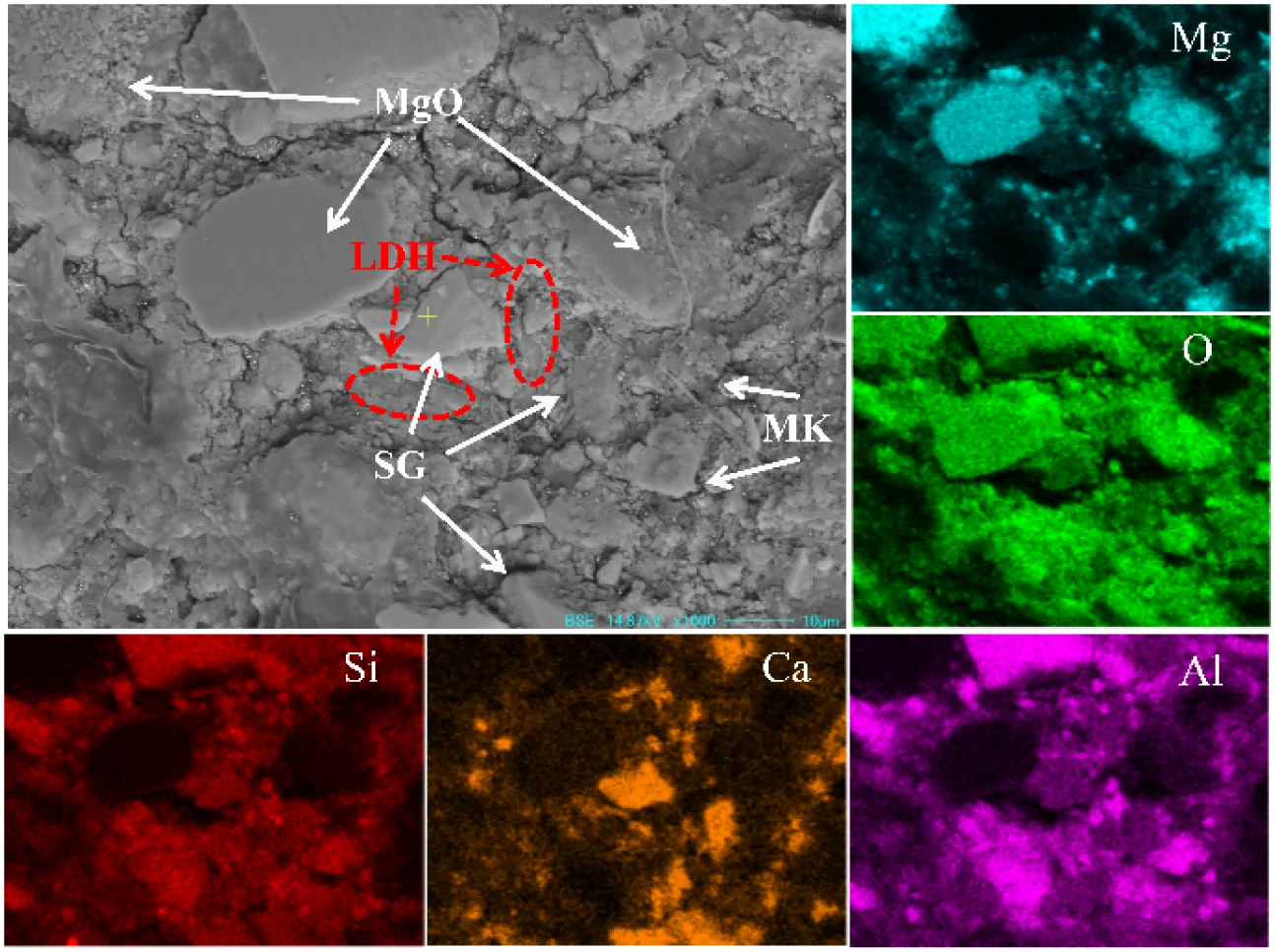
Backscattered-electron (BSE) images and elemental mapping of MMKS cementitious materials.

The BSE images show the presence of unhydrated MgO particles (irregular dark gray regions) and unreacted MK particles (bright white regions). SG particles mostly exhibit plate-like or angular morphologies, and a large number of lamellar or petal-like newly formed phases can be observed at and around their edges. Elemental mapping indicates that these regions are enriched in Al and Mg. Combined with the LDH phase information detected in the aforementioned XRD and TG analyses, these products can be identified as LDH phases. This feature represents a typical microstructural signature of the SG-containing systems.

The formation and growth of the LDH phase, together with gel products formed from the hydration of MK, SG, and MgO, significantly fill the micro-pores. The identification of LDH in these regions is supported by the combined evidence from XRD (characteristic peak at 11.2°), TG/DTG (dehydroxylation in the 400–550 °C range), and ^27^Al NMR (Al­(VI) resonance at ∼9 ppm), rather than by elemental mapping alone. While BSE-EDS mapping confirms the enrichment of Al and Mg in the lamellar regions, it alone cannot unambiguously distinguish LDH from M–A–S–H gel due to their similar elemental compositions. Nevertheless, the complementary evidence from XRD, TG, and NMR collectively supports the assignment of these lamellar products to the LDH phase. The co-existence of LDH and additional M–A–S–H gel further occupies the pore space of the original paste,^13^ thereby optimizing the pore structure and resulting in a denser and more homogeneous microstructure.

### 3.6 MIP

To elucidate the effect of SG on the performance of MMKS cementitious materials, mercury intrusion porosimetry (MIP) was conducted on selected samples with different MgO/MK ratios and SG contents after 28 days of curing. The pore size distribution curves and key parameters are presented in Figure 11 and Table 5. The MIP analysis focused on the M4K6SG20, M4K6SG40, M6K4SG20, and M6K4SG40 compositions. The M6K4SG10 composition, despite exhibiting the highest compressive strength, was not included in the MIP analysis because the primary objective of this study is to elucidate the deterioration mechanism associated with excessive SG incorporation (20% vs. 40%), rather than to simply identify the optimal formulation. Comparison between M6K4SG20 and M6K4SG40 allows direct assessment of the dilution effect at a high MgO/MK ratio, while comparison between M4K6 and M6K4 at the same SG content reveals the effect of MgO availability on pore structure regulation. Previous studies on alkali-activated metakaolin-slag systems have shown that the influence of slag content on pore structure is non-monotonic, with excessive slag incorporation leading to significant deterioration in volume stability and pore structure despite strength gains.^45^ In the present MMKS system, the transition from 20% to 40% SG captures this critical deterioration threshold, where MIP data reveal that harmful pore (>100 nm) fractions increase from 12.3% to 67.09% and the most probable pore diameter coarsens from 13.66 to 362.76 nm. Additionally, the M4K6SG0 and M6K4SG0 samples were excluded because the pore structures of the binary MgO–MK system have been previously characterized in detail,^16^ and the focus of this work is specifically on the role of SG addition.

**Figure 11:**
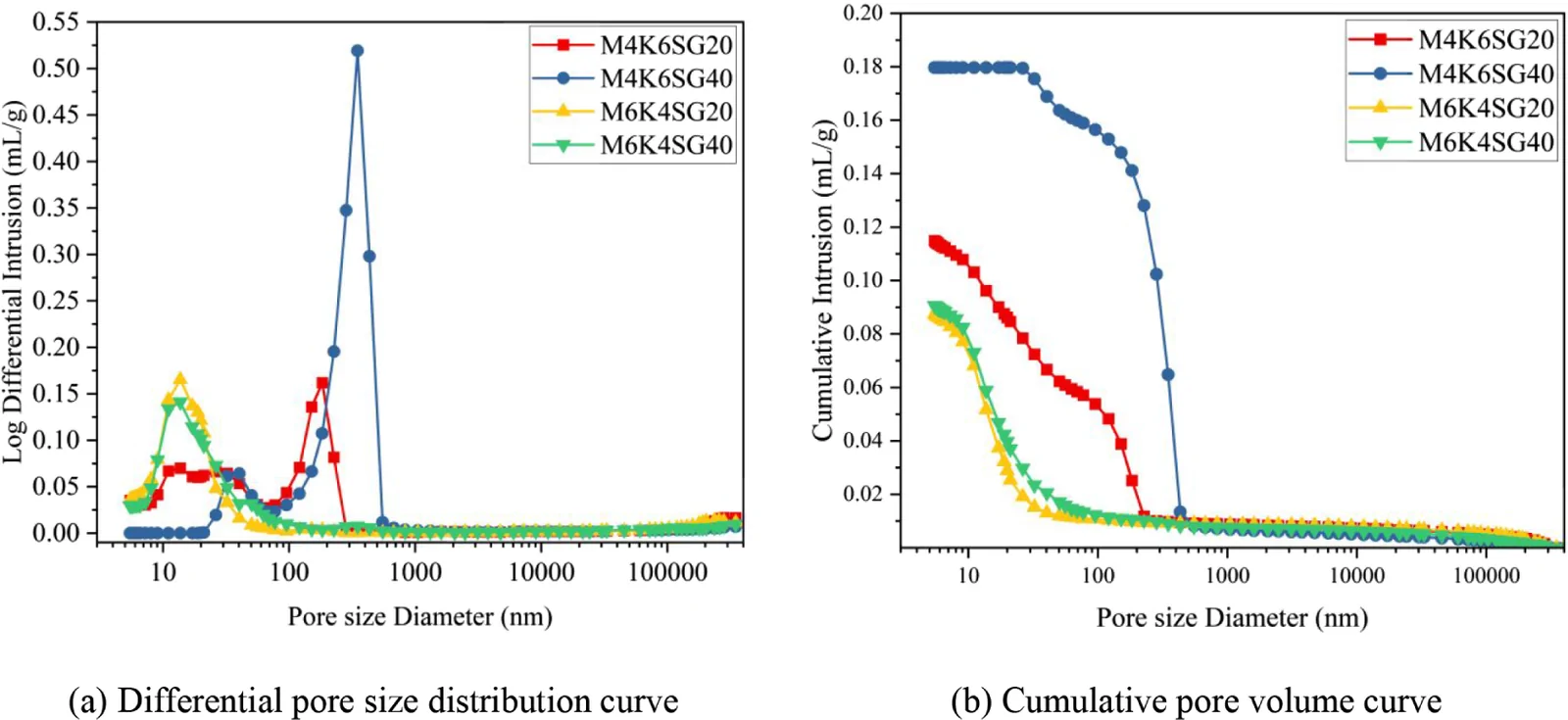
Differential and cumulative pore-size distribution curves of MMKS cementitious materials.

**Table 5:** Pore Structure Parameters of MMKS Cementitious Materials Obtained from MIP Analysis

				**Porosity of different types of pores (%)**		
**Sample**	**Average Pore Diameter (nm)**	**Most Probable Pore Diameter (nm)**	**Porosity (%)**	**>100 nm**	**10–100 nm**	**<10 nm**
M4K6SG20	29.87	183.90	20.7758	46.77	47.01	6.22
M4K6SG40	32.49	362.76	24.6937	67.09	32.91	0
M6K4SG20	15.26	13.66	16.4948	12.30	75.65	12.05
M6K4SG40	17.16	14.19	17.1647	13.58	77.19	9.01

The results indicate that the improvement of the pore structure by SG strongly depends on the MgO/MK ratio and SG content, and the observed trends are fully consistent with the mechanical strength development and previous microstructural analyses. For samples with the M4K6 mix ratio, SG incorporation led to a significant deterioration of the pore structure. At 20% SG content, the structure exhibited a highly porous characteristic, with harmful pores (>100 nm) accounting for 46.77%, the most probable pore diameter reaching 183.90 nm, and gel pores (<10 nm) favorable for strength comprising only 6.22%. When the SG content increased to 40%, the structure deteriorated further: gel pores decreased to 0%, harmful pores increased to 67.09%, and the most probable pore diameter sharply increased to 362.76 nm.

In contrast, for samples with the M6K4 mix ratio, SG significantly optimized the pore structure. At 20% SG content, the pore structure was markedly improved, with harmful pores comprising only 12.3%, the most probable pore diameter refined to 13.66 nm, and gel pores accounting for 12.05%. Even at 40% SG content, the pore parameters remained at relatively favorable levels, with harmful pores at 13.58%, gel pores at 9.01%, and the most probable pore diameter at 14.19 nm.

These observations corroborate the previously discussed compressive-strength results. In M4K6 systems, the reactivity of MK and SG is difficult to fully activate, resulting in insufficient formation of C–(A)–S–H and M–(A)–S–H gels and LDH phases to optimize the pore structure, while the dilution of active cementitious components (MgO and MK) leads to a lower total number of hydration products. Consequently, pores remain unfilled, causing coarsening and connectivity of the pore networks. By contrast, under high MgO/MK ratio conditions, MK and SG participate effectively in the reaction, promoting the formation of partial C–(A)–S–H, abundant M–(A)–S–H gels, and LDH phases (as confirmed by XRD, NMR, and BSE analyses). These products efficiently fill and refine the pores, enabling the microstructure to remain relatively dense even at high SG contents, which directly explains the excellent macroscopic mechanical performance.

### 3.7 Hydration Mechanism

The hydration of MMKS cementitious materials is a multi-stage process involving the synergistic interaction of multiple components. In the early stage of reaction, reactive MgO dissolves first, releasing Mg^2+^ and OH^–^ ions. When the ion concentration in the solution reaches the solubility product (K-sp) of Mg­(OH)_2_, Mg­(OH)_2_ precipitates and maintains the pore solution at an alkaline pH of approximately 10.5.^8^ This alkaline environment is critical for activating the dissolution of all aluminosilicate raw materials: it not only promotes the rapid cleavage of Si–O–Si and Si–O–Al bonds in MK, generating highly reactive silicate and aluminate monomers and oligomers (e.g., Si­(OH)_4_ and Al­(OH)_4_^–^), but also, under the synergistic effect of Mg^2+^ and high pH, facilitates the rapid and thorough dissolution of SG,^44^ releasing significant amounts of Ca^2+^, Al^3+^, and reactive silicate species, thereby markedly altering the ionic composition and chemical equilibrium of the system.

As the concentrations of ions and oligomers increase, complex polymerization reactions occur. In the presence of SG, two prominent polymerization pathways can be identified: first, OH^–^ continuously released from MgO hydrolysis reacts with CaO and SiO_2_ present in SG to form calcium-containing C–(A)–S–H-type oligomers; second, the high concentrations of Mg^2+^ and Al^3+^ tend to directly assemble into layered double hydroxides (LDH) based on [Mg­(OH)_6_] and [Al­(OH)_6_] octahedra.

The hydration products exhibit remarkable diversity and synergistic coexistence: a substantial portion of Al^3+^ enters six-coordinated environments, rapidly crystallizing to form the hydrotalcite-like (LDH) phase; meanwhile, the silicate network in the system undergoes significant polymerization, forming highly polymerized M–S–H/M–(A−)­S–H gel networks that interweave and coexist with LDH,^34^ as illustrated in Figure 12.

**Figure 12:**
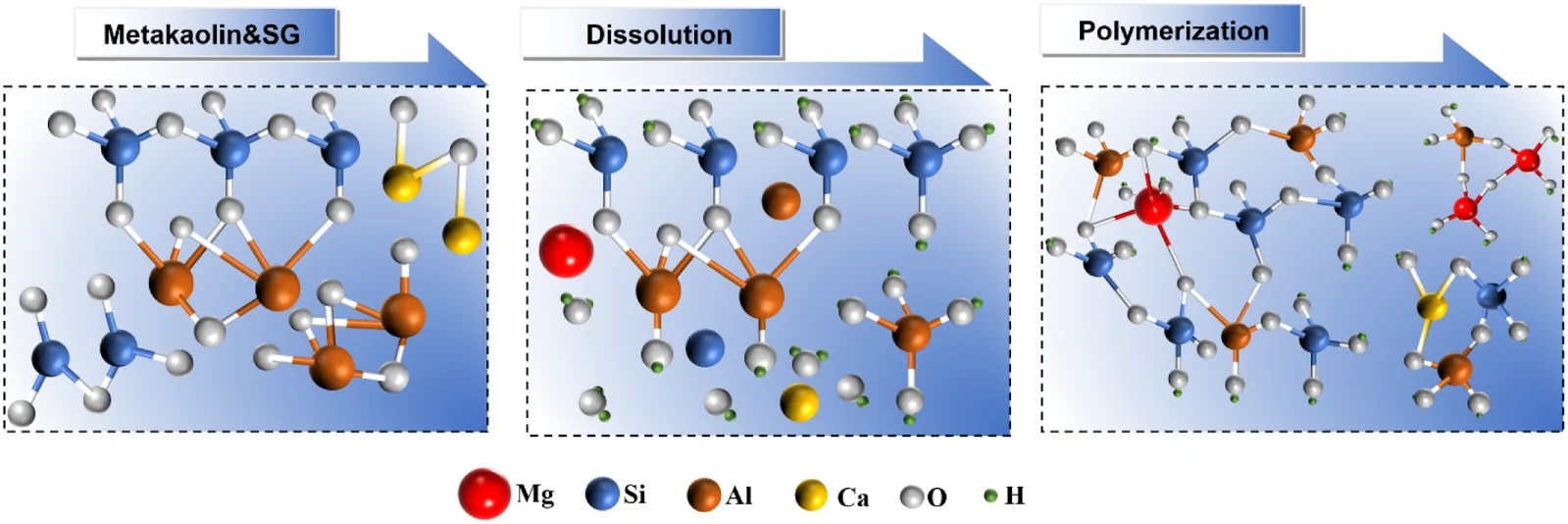
Schematic illustration of the hydration and reaction mechanism of MMKS cementitious materials.

The quantitative correlation between microstructural evolution and mechanical performance can be further elucidated by synthesizing the NMR and MIP data into a comprehensive framework. For the M6K4SG20 sample, the fraction of gel pores (< 10 nm) reaches 12.05%, with the most probable pore diameter refined to 13.66 nm, whereas the M4K6SG20 sample exhibits only 6.22% gel pores and a coarsened most probable pore diameter of 183.90 nm. Meanwhile, ^27^Al NMR quantification reveals that the Al­(VI) content in M6K4SG20 at 28 days (57.34%) and 90 days (66.35%) is substantially higher than in M4K6SG20 (48.30% at 28 days and 56.03% at 90 days), indicating a greater extent of LDH formation that contributes to pore filling. Furthermore, ^29^Si NMR-derived polymerization indicators—the *Q* ^2^/(*Q* ^1^+*Q* ^2^) ratio (0.786 for M6K4SG90 vs. 0.604 for M4K6SG28) and the mean chain length (9.36 vs. 5.04)—demonstrate a significantly higher degree of silicate network polymerization in the M6K4 system, which directly contributes to the formation of a more cohesive and load-bearing gel structure. The combined quantitative evidence from ^27^Al NMR (LDH formation), ^29^Si NMR (gel polymerization), and MIP (pore refinement) demonstrates that the enhancement in compressive strength is quantitatively linked to three interrelated factors: (1) the refinement of pore structure (reduction in most probable pore diameter from 183.90 to 13.66 nm), (2) the increased formation of crystalline LDH phases (increase in Al­(VI) content from 48.30% to 57.34% at 28 days), and (3) the enhanced polymerization of the M–(A)–S–H gel network (increase in the *Q* ^2^/(*Q* ^1^+*Q* ^2^) ratio from 0.604 to 0.786). These three factors together reduce total porosity, shift the pore size distribution toward finer capillary and gel pores, and improve the intrinsic mechanical integrity of the binder. The superior performance of the M6K4 systems thus originates from the synergistic effects of gel network polymerization (as evidenced by ^29^Si NMR) and LDH crystallization (as evidenced by ^27^Al NMR and XRD), which collectively construct a composite gel-crystalline structure with a refined pore system.

The observed strength reduction at excessive SG dosages (greater than 30%) can be attributed to a dilution effect. While SG participates in the reaction under the alkaline environment provided by MgO hydrolysis, its reactivity is lower than that of MgO. Excessive SG incorporation reduces the relative proportion of the more reactive components (MgO and MK) in the binder system, thereby decreasing the total amount of hydration products (M–(A)–S–H and LDH) formed per unit volume. Consequently, a larger fraction of unreacted or partially reacted SG particles remains embedded in the matrix, acting as weak interfaces rather than as load-bearing phases. This is consistent with the MIP results, which show significant pore coarsening at 40% SG content, particularly in the M4K6 systems where insufficient alkalinity fails to fully activate the excess slag. Therefore, the introduction of SG fundamentally alters the reaction pathways and product composition, transforming the system from a relatively homogeneous gel network to a composite system in which gel and crystalline phases (LDH) coexist. This has a profound influence on the subsequent microstructural evolution and the development of the macroscopic mechanical properties.

The key advancement of this work is the discovery that slag activation in the MMKS system is highly dependent on the MgO/MK ratio—only under high MgO/MK mass-ratio conditions does slag effectively release Al^3+^ to form LDH and enhance silicate polymerization, as evidenced by ^27^Al and ^29^Si NMR. This mechanism, previously unreported for ternary MMKS systems, provides a rational basis for designing high-performance magnesia-based binders using industrial by-products.

While the present study has elucidated the hydration mechanisms of MMKS systems through comprehensive phase and microstructural characterization, the early-age hydration kinetics (e.g., heat-flow evolution, cumulative heat release) have not been systematically characterized due to experimental constraints. Future work employing isothermal calorimetry would be valuable to quantitatively establish the relationship among the dissolution of MgO, the pozzolanic reaction of MK, and the alkali activation of SG at the earliest reaction stages, thereby providing a more complete picture of the hydration process.

## 4 Conclusions

In this study, the incorporation of slag (SG) into the MgO–MK binary cementitious system was systematically investigated. The main conclusions are as follows:

(1) Optimal SG content and workability: the optimal SG incorporation range is approximately 10–20% under a high MgO/MK ratio (M6K4). At 20% SG, the fluidity of the M6K4 paste increased by 21.61% compared with the SG-free sample. Hydration heat analysis further confirms that the M6K4SG20 specimen exhibits the highest cumulative heat release among all formulations, with a ranking of M6K4SG20 > M4K6SG20 > M6K4SG40 > M4K6SG40, indicating that a moderate SG content (20%) combined with a high MgO/MK ratio provides the most favorable hydration kinetics and late-age reaction potential. In contrast, excessive SG (40%) lowers the total heat release due to a dilution effect that reduces the effective content of reactive MgO and MK per unit mass. Excessive SG (>30%) also leads to strength reduction due to dilution of active components.
(2) Reinforcement mechanism: SG can be effectively activated at a proper MgO/MK ratio (M6K4), providing active Al^3+^ that promotes both the polymerization of silicate networks (M–A–S–H gel) and the formation of hydrotalcite-like (LDH) phases. ^27^Al NMR confirms that Al^3+^ enters the LDH in a six-coordinated configuration. The composite structure of the M–A–S–H gel interwoven with LDH crystals underlies the enhanced mechanical performance.
(3) Pore structure regulation: MIP analysis demonstrates that SG optimizes the pore structure only at an appropriate MgO/MK ratio (M6K4), where LDH and M–A–S–H effectively fill capillary pores and increase the fraction of gel pores (< 10 nm). At the M4K6 ratio, the SG addition leads to pore coarsening due to the dilution effect and insufficient hydration products.

In summary, SG can significantly enhance the performance of the MgO–MK system when it is applied at suitable ratios and contents. This study clarifies the hydration and reinforcement mechanisms of MMKS cementitious materials, providing a theoretical basis for the design of high-performance magnesium-based binders using industrial solid wastes.

## Data Availability

The datasets generated during and/or analyzed during the current study are available from the corresponding author on reasonable request.
